# Cluster-Based Pairwise Contrastive Loss for Noise-Robust Speech Recognition

**DOI:** 10.3390/s24082573

**Published:** 2024-04-17

**Authors:** Geon Woo Lee, Hong Kook Kim

**Affiliations:** 1 AI Graduate School, Gwangju Institute of Science and Technology, Gwangju 61005, Republic of Korea; geonwoo0801@gist.ac.kr; 2 School of Electrical Engineering and Computer Science, Gwangju Institute of Science and Technology, Gwangju 61005, Republic of Korea; 3 AunionAI Co., Ltd., Gwangju 61005, Republic of Korea

**Keywords:** joint training, noise-robust speech recognition, speech enhancement, contrastive loss, self-supervised learning, acoustic tokenizer

## Abstract

This paper addresses a joint training approach applied to a pipeline comprising speech enhancement (SE) and automatic speech recognition (ASR) models, where an acoustic tokenizer is included in the pipeline to leverage the linguistic information from the ASR model to the SE model. The acoustic tokenizer takes the outputs of the ASR encoder and provides a pseudo-label through K-means clustering. To transfer the linguistic information, represented by pseudo-labels, from the acoustic tokenizer to the SE model, a cluster-based pairwise contrastive (CBPC) loss function is proposed, which is a self-supervised contrastive loss function, and combined with an information noise contrastive estimation (infoNCE) loss function. This combined loss function prevents the SE model from overfitting to outlier samples and represents the pronunciation variability in samples with the same pseudo-label. The effectiveness of the proposed CBPC loss function is evaluated on a noisy LibriSpeech dataset by measuring both the speech quality scores and the word error rate (WER). The experimental results reveal that the proposed joint training approach using the described CBPC loss function achieves a lower WER than the conventional joint training approaches. In addition, it is demonstrated that the speech quality scores of the SE model trained using the proposed training approach are higher than those of the standalone-SE model and SE models trained using conventional joint training approaches. An ablation study is also conducted to investigate the effects of different combinations of loss functions on the speech quality scores and WER. Here, it is revealed that the proposed CBPC loss function combined with infoNCE contributes to a reduced WER and an increase in most of the speech quality scores.

## 1. Introduction

The recent developments in neural network architecture and training approaches have facilitated continuous progress, which has manifested in enhanced capabilities in terms of automatic speech recognition (ASR) [1,2]. The current state-of-the-art ASR systems are approaching the levels of human recognition in terms of performance [3] and are ready to be deployed in applications such as voice-based information retrieval, chatbots, and automated transcription systems [4]. Moreover, there is an increasing interest in ASR operating in real-world scenarios for human–robot interactions within industry [5] and dialogue systems for social safety [6]. However, ASR models often experience performance degradation in distant microphone settings or under conditions with a low signal-to-noise ratio (SNR), due to the distortion of the speech signals by real-world ambient noise [7,8].

To improve ASR performance in noisy environments, multi-condition training (MCT) and noise-aware training (NAT) techniques have been studied, using noise as a condition [9,10]. However, unseen noise or unpredicted variations in noise can limit the ASR performance, even when MCT and NAT techniques are applied. To address this limitation, speech enhancement (SE) models, employed as preprocessors for the ASR model, have been developed to suppress the noise and provide enhanced speech [11,12,13,14]. However, these SE models can introduce unintended artifacts into the enhanced speech signal, which can create an additional form of mismatching in ASR, degrading its performance [13,14]. To remedy this mismatching problem, speech signals enhanced by the SE model are added to the training dataset, meaning that the artifacts in the enhanced speech are trained in a multi-condition framework. Nevertheless, the improvements in the ASR performance obtained through the MCT approach are limited, because the artifacts in the enhanced speech remain unpredictable [9].

As an alternative, a pipeline integrating SE and ASR models has been explored in a joint training framework [9,15], where an SE model is used as the front-end of the ASR model. Although jointly training the pipeline leads to a better ASR performance than when using the MCT approach [9], difficulties can occur due to the conflicting gradients between the SE and ASR models, resulting in a convergence issue, which is referred to as a conflicting problem [16,17]. The conflicting gradients originate from the different gradient scales and directions between the SE and ASR models, which is caused by the differences in their neural architectures and loss functions, with different task goals. To solve the conflicting problem, several training approaches have been studied, including those based on asynchronous subregion optimization (ASO) [18,19], gradient surgery [20,21], and knowledge distillation (KD) [22,23,24].

Among these approaches, KD-based training achieves the best ASR performance by adjusting the gradient scales and directions of both the SE and the ASR models. In other words, the SE model is trained using a loss function that is defined in the middle layer of the ASR model. Therefore, the gradients of the SE model have more positive directions and closer scales than when the SE loss function is defined in the output layer of the ASR model. For example, the output feature vectors from an acoustic model, which is the initial part of the ASR model, are clustered, and the SE model is then trained using the cross-entropy (CE) loss to predict the centroid from the clustering [23]. Instead of directly using ASR, or a part of the ASR model, the loss function for the SE model is designed as the CE loss between the quantized vectors of clean and enhanced speech signals from the Wav2Vec 2.0 pretrained model [24,25]. However, the use of these targets in the CE loss could result in performance degradation, due to overfitting on hard examples [26,27]. To mitigate this problem, metric learning using the supervised contrastive (SupCon) loss [28], which is effective in feature representation and uses pairwise distances, can be employed for image classification [27]. However, applying the SupCon loss requires target labels, whereas the joint training proposed in this paper should be successful without target labels.

Therefore, this paper proposes the cluster-based pairwise contrastive (CBPC) loss, which is a self-supervised version of the SupCon loss, to train a pipeline comprising SE and ASR models in order to achieve an improvement in the ASR performance. First, the ASR model is trained using a training dataset and then frozen, as it will be used to transfer the linguistic information to the SE model. Subsequently, the output vectors of the ASR encoder are clustered through K-means clustering for the transfer process, where the cluster indices are referred to as pseudo-labels in this paper. Finally, the proposed CBPC loss function using the pseudo-labels is applied to the SE model training. The contributions of this paper can be summarized as follows:

- The CBPC loss function is proposed to leverage the linguistic information for the SE model by extending the SupCon loss to a self-supervised version. Replacing the CE loss, the proposed CBPC loss is used to train the pipeline with pseudo-labels. Accordingly, the proposed CBPC loss contributes to preventing the SE model from overfitting to the outlier samples in each cluster, resulting in an improved ASR performance compared to that of the CE loss.
- To further improve the ASR performance, the proposed CBPC loss is combined with the information noise contrastive estimation (infoNCE) loss [29] to train the SE model to represent the intra-cluster pronunciation variability. This is because the proposed CBPC loss function focuses on increasing the inter-cluster representation ability. Therefore, the combined loss also contributes to retaining the contextual information among the utterances with the same pseudo-label.
- An ablation study is conducted to examine the contributions of different combinations of loss functions to the SE and ASR performance.

The remainder of this paper is organized as follows: Section 2 presents a brief review of the methodologies of the joint training approaches applied to a pipeline comprising SE and ASR models. Section 3 proposes the CBPC loss function to train the SE model in the pipeline for an improved ASR performance. Subsequently, Section 4 explains the experimental setup and evaluation metrics. Then, Section 5 evaluates the performance of the SE and ASR models trained by the proposed loss function on the noisy LibriSpeech dataset by measuring both the speech quality scores and the word error rate (WER). In addition, the performance of the SE and ASR models trained using the proposed training approach is compared with those of models trained using conventional joint training approaches. Moreover, an ablation study is conducted to discuss the SE and ASR performances according to the different combinations of loss functions applied in the proposed training approach. Finally, Section 6 concludes the paper.

## 2. Pipeline Comprising SE and ASR for Noise-Robust ASR

**Figure 1:**
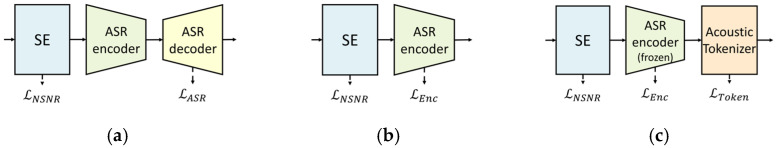
Block diagrams of a pipeline comprising speech enhancement (SE) and automatic speech recognition (ASR) models: (**a**) a joint training approach using the information on the ASR decoder, (**b**) a joint training approach using the information on the middle layer (ASR encoder) of the ASR model, and (**c**) the proposed joint training approach using an acoustic tokenizer.

A conventional pipeline comprising SE and ASR models for joint training is illustrated in Figure 1a [19,20,21,22]. Conventional joint training approaches combine all possible losses, such as the negative SNR (NSNR) loss ($L_{SE}$) and ASR loss ($L_{ASR}$), and they jointly or asynchronously train the pipeline [19,20,21,22]. However, the SE and ASR models have the following different goals: the prediction of clean speech and word sequences, respectively. Thus, there exist conflicting gradients, due to the different gradient directions of the two losses. Figure 1b shows a pipeline for the training of the SE model using a loss function calculated from the middle layer of the ASR model, i.e., the ASR encoder [24,25]. Compared with the pipeline depicted in Figure 1a, the gradients of the loss function are closer to those of the NSNR loss in the pipeline shown in Figure 1b.

In addition, instead of directly using the middle layer of the ASR model, a specifically designed layer can be added to represent the outputs of the ASR model to train the SE model with less conflicting gradients. In this paper, an acoustic tokenizer is designed to leverage the linguistic information derived from the ASR encoder and transfer this information to the SE model [23]. Figure 1c illustrates this pipeline for the training of the SE model, which is achieved by concatenating an acoustic tokenizer to the ASR encoder. In contrast to the ASR encoder shown in Figure 1b, this acoustic tokenizer serves as a surrogate model capable of extracting linguistic information at frame-wise granularity.

To train the acoustic tokenizer, the output vector of the ASR encoder is used as an input feature for K-means clustering. Subsequently, the cluster indices are utilized as pseudo-labels to calculate the proposed CBPC loss. In fact, three different losses are computed, as follows: the NSNR loss, $L_{NSNR}$; the ASR encoder loss, $L_{Enc}$; and the acoustic tokenizer loss, $L_{Tokenizer}$. Finally, the SE model is trained through backpropagation using these losses. In this paper, the deep complex convolution neural network (DCCRN)-based SE model and conformer (encoder)–transducer (decoder)-based ASR model are employed. For a fair comparison, the architecture and hyperparameters of these models are set identically to those in [30,31], respectively.

## 3. Proposed Cluster-Based Pairwise Contrastive Loss Function for Joint Training

This section explains the training procedure of the SE model from the ASR encoder combined with the acoustic tokenizer. To distillate the linguistic information from the ASR encoder to the SE model, the conformer–transducer-based ASR model is first trained and then fixed. Subsequently, the acoustic tokenizer is trained by the proposed CBPC loss function using clean speech signals from the training dataset used for the ASR model training. Next, the SE model is trained using a set of clean utterances and their noisy version by applying the three losses described in Figure 1c. Next, the main components of the pipeline (the acoustic tokenizer and the loss functions) are described in detail.

### 3.1. Acoustic Tokenizer

**Figure 2:**
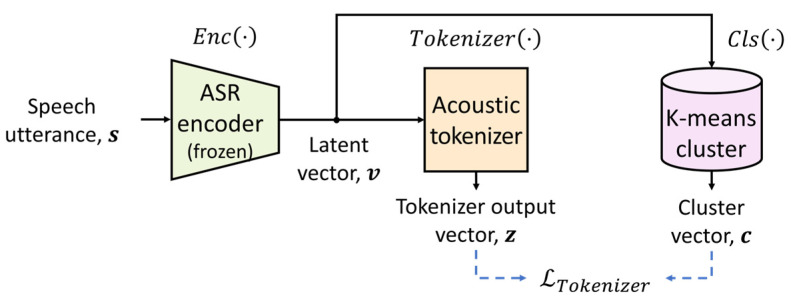
Block diagram of the training procedure of the acoustic tokenizer.

Figure 2 depicts the training procedure of the acoustic tokenizer using clean speech utterances from the training dataset, where the ASR encoder is frozen, as mentioned previously. Given a dataset, $s={\left\{s_{n}\right\}}_{n=1,\cdots ,N}$, composed of clean speech utterances with a mini-batch size N, each utterance is sampled at 16 kHz and segmented into consecutive frames of 25 ms in length, with an overlap length of 16 ms, resulting in $s={\left\{s_{n,t}\right\}}_{n=1,\cdots ,N,t=1,\cdots ,T}$. Here, $D_{f}=400$ and T is the total number of frames in s. Then, s is input into the ASR encoder, $Enc\left(\cdot \right),$ yielding the output sequence $v=Enc\left(s\right)={\left\{v_{n,m}\right\}}_{n=1,\cdots ,N,m=1,\cdots ,M},$ where $v_{n,m}(\in R^{D_{e}})$ is the m-th latent vector with a dimension of $D_{e}(=144)$. To speed up the training and inference, $Enc\left(\cdot \right)$ employs subsampling layers to reduce the frame rate by a factor of 4, meaning that $M=\left\lfloor T/4\right\rfloor$.

Each $v_{n,m}$ is coded using a K-means clustering algorithm, $Cls\left(\cdot \right),$ resulting in a one-hot cluster vector, $c_{n,m}=Cls\left(v_{n,m}\right)\in {\left\{0,1\right\}}^{D_{c}}$, which is referred to as a pseudo-label of $v_{n,m}$. Herein, the number of clusters, $D_{c},$ is set to 1.5 k, because the ASR model trained in this work includes the 1 k of linguistic units generated by the unigram language algorithm. In addition to these units, acoustic noise (such as breathing and coughing) is included in the training dataset. To obtain the K-means clusters, the mini-batch K-means algorithm in the scikit-learn [32] package is applied to the pool of v, which is obtained from all of the clean speech utterances in the training dataset. Furthermore, the latent vectors corresponding to the silent frames are removed. These silent frames are detected by applying a voice activity detection technique to clean utterances with a 40-dB cutoff amplitude level [33].

Next, v is tokenized into z, such as $z=Tokenizer\left(v\right)={\left\{z_{n,m}\right\}}_{n=1,\cdots ,N,m=1,\cdots ,M}\in R^{D_{c}}$, where $Tokenizer\left(\cdot \right)$ is constructed via a one-time-distributed layer. To train the tokenizer, the acoustic tokenizer loss function, $L_{Tokenizer}\left(z|c\right)$, is defined using the tokenizer output vectors, $\left\{z_{n,m}\right\}$, and cluster vectors, $\left\{c_{n,m}\right\}$, as follows:

(1) $$L_{Tokenizer}\left(z|c\right)=-\sum_{n=1}^{N}\sum_{m=1}^{M}\log\left(\frac{\exp\left(z_{n,m,i}/\tau_{a}\right)}{\sum_{j=1}^{D_{c}}\exp\left(z_{n,m,j}/\tau_{a}\right)}\right)$$

where $z_{n,m,i}$ is the *i*-th element of $z_{n,m}$ at which $c_{n,m,\;i}$ is 1 and $\tau_{a}\left(=0.5\right)$ denotes the temperature parameter.

### 3.2. Contrastive Learning for Acoustic Tokenizer

The use of contrastive loss in metric learning facilitates the attraction of positive/negative pairs, and it has demonstrated notable performance improvements over CE loss across various domains [28,34]. The rationale behind this result is that, while the CE loss might be overfitted to hard samples, contrastive loss, which is grounded on the distance between the positive and negative pairs, mitigates the optimization issue associated with specific samples [26,27]. In addition to metric learning, contrastive loss has gained prominence in the realm of self-supervised learning, exhibiting an exemplary performance in the speech domain, such as contrastive predictive coding (CPC) [29] and Wav2vec 2.0 [25].

However, an inherent challenge in feature representation learning through contrastive loss is the potential convergence to a trivial constant solution [35,36]. To address this issue, the spread loss [37] leverages the supervised contrastive (SupCon) loss [28] with the information noise contrastive estimation (infoNCE) loss, which incorporates a regularization term to prevent the representation from collapsing to a singular point [29]. In essence, while the SupCon loss encourages attraction within the same class, the infoNCE loss induces repulsion, effectively resolving the collapsed representation dilemma and ensuring successful feature representation. However, to apply the SupCon loss in this joint training approach, target labels are required, because the SupCon loss is designed in a supervised learning framework.

**Figure 3:**
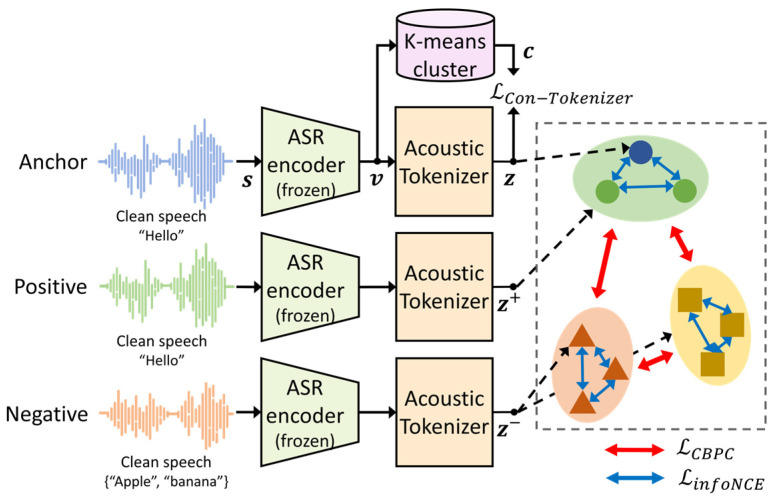
Training procedure of the acoustic tokenizer using the proposed cluster-based pairwise contrastive (CBPC) loss function.

Therefore, this paper proposes CBPC loss, which is a self-supervised version of the SupCon loss, by using a clustering technique. The training procedure of the acoustic tokenizer using the proposed CBPC loss for noise-robust ASR is illustrated in Figure 3. First, a latent vector, $v_{n,m}$, at the *n*-th mini-batch and *m*-th frame, is clustered into $c_{n,m}$, which is then used as a pseudo-label for $v_{n,m}$, as described in Section 3.1. Next, $v_{n,m}$ is tokenized into $z_{n,m}$, and a set of the positive pairs for $z_{n,m}$ is defined as a set with the same pseudo-label, defined as $P\left(z_{n,m}|c\right)=\left\{z_{n,l}\;|\;Cls\left(v_{n,l}\right)=c_{n,m},\;l=1,\ldots ,M\right\}$. Otherwise, a set of negative pairs for $z_{n,m}$ is defined as $N\left(z_{n,m}|c\right)=\left\{z_{n,l}\;|\;Cls\left(v_{n,l}\right)\neq c_{n,m},\;l=1,\ldots ,M\right\}.$ Then, the proposed CBPC loss function for the acoustic tokenizer conditioned by c, $L_{CBPC}\left(z|c\right)$, is defined as follows:

(2) $$L_{CBPC}\left(z|c\right)=\sum_{n=1}^{N}\sum_{m=1}^{M}\frac{-1}{\left|P\left(z_{n,m}|c\right)\right|}\sum_{z_{n,m}^{+}\in P\left(z_{n,m}|c\right)}\log\frac{\exp\left(z_{n,m}\cdot z_{n,m}^{+}/\tau_{c}\right)}{\sum_{z_{k,l}\;\in \left\{P\left(z_{n,m}|c\right),N\left(z_{n,m}|c\right)\backslash \{z_{n,m}^{+}\}\right\}}\exp\left(z_{n,m}\cdot z_{k,l}/\tau_{c}\right)}$$

where $\tau_{c}\left(=0.5\right)$ denotes the temperature of the proposed CBPC loss function and $\left|P\left(z_{n,m}|c\right)\right|$ is its cardinality. As shown in Equation (2), the CBPC loss function aims to maximize the distance between the clusters.

By only applying the CBPC loss in Equation (2), the tokenizer output vectors, z, can be overly drawn toward the centroid, which can result in the loss of contextual information [26,27]. Such a phenomenon could subsequently result in a degraded ASR performance. To remedy this issue, the infoNCE loss [29] is incorporated here to ensure repulsion within the intra-cluster, where all of the samples in the same cluster, except itself, are treated as negative samples. Specifically, the infoNCE loss function can be defined as follows [29]:

(3) $$L_{infoNCE}\left(z|c\right)=-\sum_{n=1}^{N}\sum_{m=1}^{M}\log\frac{\exp\left(z_{n,m}\cdot z_{n,m}/\tau_{c}\right)}{\sum_{z_{k,l}\;\in P\left(z_{n,m}|c\right)}\exp\left(z_{n,m}\cdot z_{k,l}/\tau_{c}\right)}.$$

Subsequently, the final contrastive acoustic tokenizer loss function used to train the acoustic tokenizer combines the acoustic tokenizer loss in Equation (1) and CBPC loss in Equation (2) with the infoNCE loss in Equation (3), which is defined as follows:

(4) $$L_{Con-Tokenizer}\left(z|c\right)=\theta \cdot L_{Tokenizer}\left(z|c\right)+\left(1-\theta \right)\left(\delta \cdot L_{CBPC}\left(z|c\right)+\left(1-\delta \right)\cdot L_{infoNCE}\left(z|c\right)\right)$$

where θ controls the weighting between the acoustic tokenizer and the contrastive losses and δ gives different weights to the proposed CBPC loss and infoNCE loss. The weights, θ and δ, in Equation (4), are determined according to the following procedure: First, θ is fixed at 0.5 and δ is varied in steps of 0.1 from 0.1 to 1.0. The acoustic tokenizer is trained at each step using Equation (4), where the validation dataset in the LibriSpeech dataset is used. Then, the classification accuracy of the trained acoustic tokenizer is calculated by comparing $z_{n,m}$ and $c_{n,m}$, and the δ with the highest accuracy is selected. This process is repeated by varying θ with a fixed δ to select the best value of θ. As a result, θ and δ are set to 0.7 and 0.9, respectively, and the model parameters of the acoustic tokenizer trained with these weights are fixed to train the SE model.

### 3.3. SE Model Training

**Figure 4:**
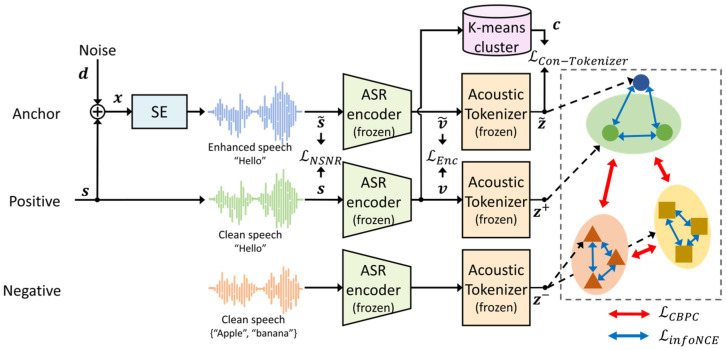
Block diagrams of the proposed joint training approach for the SE model using a negative signal-to-noise ratio (NSNR), an ASR encoder, and the contrastive acoustic tokenizer loss function.

Figure 4 displays the training procedure of the SE model using three different loss functions, where the ASR encoder and the acoustic tokenizer are fixed, as mentioned previously. To train the SE model using the information on the ASR encoder through contrastive learning, noisy utterances are generated by mixing a noise signal, d, with s, such that $x=s+d{=\left\{x_{n,t}\right\}}_{n=1,\cdots ,N,t=1,\cdots ,T}$. As shown in the figure, x is passed into the SE model, which is randomly initialized, to predict the estimated clean utterances, $\tilde{s}$. The clean and estimated clean utterances are then input into the ASR encoder to obtain the following two sequences of latent vectors: $v=Enc\left(s\right)$ and $\tilde{v}=Enc\left(\tilde{s}\right)$. Then, the latent vectors are further encoded using the tokenizer, such as $z=Tokenizer\left(v\right)$ and $\tilde{z}=Tokenizer\left(\tilde{v}\right)$. Simultaneously, v is clustered as $c_{n,m}=Cls\left(v_{n,m}\right),$ as mentioned in Section 3.1.

There are three loss functions in this training approach. The speech quality loss, $L_{NSNR}\left(\cdot ,\cdot \right),$ is first computed for a given pair of clean and noisy utterances, s and $\tilde{s}$, which is defined as follows:

(5) $$L_{NSNR}\left(s,\tilde{s}\right)=-\frac{1}{N}10\log_{10}\left(\frac{{\left\|s\right\|}^{2}}{{\left\|s-\tilde{s}\right\|}^{2}}\right).$$

The second loss function is the ASR encoder loss, $L_{Enc}\left(\cdot ,\cdot \right),$ which is defined as the $L_{2}$-norm between two latent vector sequences, v and $\tilde{v}$, from s and $\tilde{s}$, as follows:

(6) $$L_{Enc}\left(v,\;\tilde{v}\right)=\frac{1}{N}{\left\|v-\tilde{v}\right\|}^{2}.$$

Finally, the contrastive acoustic tokenizer loss function is computed using the tokenizer output vectors of $\tilde{s}$ and $\tilde{z}$, as well as the cluster vectors, c, with positive/negative pairs in the tokenizer output vector domain, $P\left(z_{n,m}|c\right)$ and $N\left(z_{n,m}|c\right)$, of s. This loss function should be conditioned by z and c, as shown in the following equation:

(7) $$\begin{array}{c} L_{Con-Tokenizer}\left(\tilde{z}|z,c\right)\\ =\theta \cdot L_{Tokenizer}\left(\tilde{z}|c\right)+\left(1-\theta \right)\left(\delta \cdot L_{CBPC}\left(\tilde{z}|z,c\right)+\left(1-\delta \right)\cdot L_{infoNCE}\left(\tilde{z}|z,c\right)\right) \end{array}$$

where θ and δ are set identically to those in Equation (4). In Equation (7), $L_{Tokenizer}\left(\tilde{z}|c\right)$ is a noisy version of Equation (1), rewritten as follows:

(8) $$L_{Tokenizer}\left(\tilde{z}|c\right)=-\sum_{n=1}^{N}\sum_{m=1}^{M}\log\left(\frac{\exp\left({\tilde{z}}_{n,m,i}/\tau_{a}\right)}{\sum_{j=1}^{D_{c}}\exp\left({\tilde{z}}_{n,m,j}/\tau_{a}\right)}\right).$$

In addition,

(9) $$L_{CBPC}\left(\tilde{z}|z,c\right)=\sum_{n=1}^{N}\sum_{m=1}^{M}\frac{-1}{\left|P\left(z_{n,m}|c\right)\right|}\sum_{z_{n,m}^{+}\in P\left(z_{n,m}|c\right)}\log\frac{\exp\left({\tilde{z}}_{n,m}\cdot z_{n,m}^{+}/\tau_{c}\right)}{\sum_{z_{k,l}\;\in \left\{P\left(z_{n,m}|c\right),N\left(z_{n,m}|c\right)\backslash \{z_{n,m}^{+}\}\right\}}\exp\left({\tilde{z}}_{n,m}\cdot z_{k,l}/\tau_{c}\right)}$$

and

(10) $$L_{infoNCE}\left(\tilde{z}|z,c\right)=-\sum_{n=1}^{N}\sum_{m=1}^{M}\log\frac{\exp\left({\tilde{z}}_{n,m}\cdot z_{n,m}/\tau_{c}\right)}{\sum_{z_{k,l}\;\in P\left(z_{n,m}|c\right)}\exp\left({\tilde{z}}_{m,n}\cdot z_{k,l}/\tau_{c}\right)}.$$

Finally, the joint loss function for SE training is obtained by combining all of the losses in Equations (5)–(7), denoted as follows:

(11) $$L=\alpha \cdot L_{NSNR}\left(s,\tilde{s}\right)+\beta \cdot L_{Enc}\left(v,\tilde{v}\right)+\gamma \cdot L_{Con-Tokenizer}\left(\tilde{z}|z,c\right).$$

where α, β, and γ are the weights of the NSNR, ASR encoder loss, and tokenizer loss, respectively. The three weights are determined by following the procedure described in [22]. Consequently, α, β, and γ are set as 0.3, 0.7, and 1.0, respectively.

## 4. Experimental Setup

In this section, the performance of the proposed training approach was evaluated for noise-robust ASR, and it was then compared with the performance of MCT and conventional joint training approaches, including asynchronous subregion optimization (ASO)-based joint optimization [18], gradients-surgery (Grad)-based joint optimization [19], and acoustic tokenizer (Token)-based joint optimization [22]. Here, the ASO-based joint optimization approach was first used to train a pipeline with the SE and ASR encoder losses, and it was then further trained with the combination of the SE and ASR losses. Meanwhile, the Grad-based joint optimization approach was used to train a pipeline with projection errors from the SE gradients to the ASR ones. The Token-based joint optimization approach was implemented similarly to the proposed training approach, but it differed in terms of the loss functions. In particular, the Token-based approach used the cross-entropy loss to train the SE model in the pipeline, while the proposed training approach used contrastive losses such as the CBPC and infoNCE losses. The ASR and SE performance was measured on a simulated noisy dataset mixed with the LibriSpeech [38] and deep noise suppression (DNS) challenge datasets [39].

### 4.1. Dataset

A total of 281,241 clean speech utterances spoken by 2338 speakers were excerpted from train-clean-100, train-clean-360, and train-other-500 in the LibriSpeech dataset to obtain the clean speech training dataset. Here, the total length of all of the utterances was 960 h, and the average length per utterance was measured as 12.3 s. To simulate various acoustic noise conditions, a noisy training dataset was constructed using the noise dataset released from the DNS dataset. This noise dataset was collected from AudioSet, Freesound, and the Diverse Environments Multi-Channel Acoustic Noise Dataset (DEMAND), which included approximately 150 different noise types. The noisy speech utterances were obtained by mixing each clean speech utterance with noise data that were randomly selected from the noise dataset. To simulate different noise levels, the mixing ratio between the clean speech and noise was controlled, ensuring that the SNR ranged from −5 to 5 dB.

To validate the model training, the dev-clean and dev-other datasets were used as validation datasets. The dev-clean and dev-other datasets were composed of 2703 and 2864 clean speech utterances, respectively. To create the noisy version of the validation dataset, DNS noise was randomly added to each of the clean utterances under an SNR in the range of −5 to 5 dB. Next, to evaluate the ASR and SE performance of the various training approaches, including the proposed training approach, the test-clean and test-other datasets in the LibriSpeech dataset were used, which contained 2620 and 2939 clean speech utterances, respectively. Similar to the validation dataset described above, the noisy version of the evaluation dataset was obtained by adding DNS noise to each of the clean utterances.

### 4.2. Hyperparameters

#### 4.2.1. Model Architecture

The architecture and hyperparameters of the DCCRN-based SE model were set identically to those used in [30]. In other words, the input feature was a complex spectrum obtained by applying a 512-point short-time Fourier transform to each noisy speech frame with a frame size of 25 ms and a frame hop size of 16 ms. The number of complex convolutional blocks for both the encoder and decoder was set to six, and these six convolutional blocks had varying numbers of channels, such as [32, 64, 128, 128, 256, 256], with a kernel size of 5 × 2 and a stride size of 2 × 1.

The architecture and hyperparameters of the conformer–transducer-based ASR model were also set identically to those of the conformer(s) described in [31]. As an input feature of the ASR model, an 80-dimensional log-mel spectrum was extracted. The ASR encoder was composed of 16 conformer blocks, and each conformer block extracted a latent vector with a dimension of 144 $\left(D_{e}\right)$. As a target feature, the linguistic units for transcribing the target texts consisted of a special token and 1k linguistic units generated by the unigram language model algorithm [40].

#### 4.2.2. Training Details and Implementation

The ASR and SE models were trained using the noisy LibriSpeech training dataset, while the tokenizer was trained using the clean LibriSpeech training dataset. In this study, the Adam optimizer was applied to all of the model training approaches. To adjust the learning rate, the warmup learning rate scheduler technique with 40,000 warmup steps was applied to train the conformer–transducer-based ASR model, while a plateau learning rate scheduler with patience of 5 and a factor of 0.5 was utilized for the acoustic tokenizer and SE model training. In particular, the SpecAugment technique was employed for the ASR model training. All of the experiments were implemented in Python 3.8.10 using TensorFlow 2.11.0 [41] conducted on an Intel(R) Xeon(R) Gold 6226R workstation using four Nvidia RTX 3090s.

## 5. Performance Evaluation and Discussion

### 5.1. Results and Discussion of ASR Performance

The ASR performance of each of the training approaches was evaluated by measuring the WER on both the validation and the test datasets. The WER of the ASR model trained using the proposed training approach was then compared to those of six different approaches, as follows: (1) an ASR model trained via the MCT using the clean and noisy training datasets (denoted as MCT-noisy); (2) an SE model trained on the clean and noisy speech training datasets, wherein the enhanced signal was subsequently fed into the MCT-noisy ASR model (denoted as MCT-noisy + standalone-SE); (3) an ASR model trained by the MCT using the clean, noisy, and enhanced data from the standalone-SE datasets (denoted as MCT-all); (4) a combination of the SE and ASR models trained by conventional joint optimization (denoted as Joint-Straight) [9]; (5) a pipeline trained by ASO-based joint optimization (denoted as Joint-ASO) [18]; (6) a pipeline trained by Grad-based joint optimization (denoted as Joint-Grad) [20]; and (7) a pipeline trained by Token-based joint optimization (denoted as Joint-Token) [22].

Table 1 compares the average WERs of the ASR models trained using different training approaches, where the performance evaluation was carried out on four different noisy datasets constructed by mixing noise with the dev-clean, dev-other, test-clean, and test-other datasets. First, the conventional training approaches were compared. As shown in the table, the average WER of the MCT-noisy + standalone-SE model was increased because the standalone-SE model unexpectedly distorted the speech. However, the standalone-SE model improved the speech quality, which will be discussed in the next subsection. Upon adding enhanced speech to the training data, the WERs of MCT-all were marginally lower than those of MCT-noisy for all datasets. This was because the mismatching issue between the training and evaluation was somewhat mitigated.

Second, the average WERs of the ASR models were compared according to the different joint training approaches. Note that the training hyperparameters for Joint-Straight, Joint-ASO, and Joint-Grad were set identically to those in the corresponding papers. As shown in the table, Joint-Token exhibited the lowest WERs among all of the joint training approaches. Furthermore, the average WER of the ASR model trained by the joint training approach using the proposed contrastive loss was relatively reduced by 15.39% compared to that of MCT-noisy. Moreover, the proposed joint training approach achieved a relative WER reduction of 3.82%, compared to Joint-Token, which had the lowest WER among the conventional joint training approaches.

### 5.2. Results and Discussion of SE Performance

The speech quality scores of the various training approaches were compared and examined by measuring the perceptual evaluation of speech quality (PESQ) [42], short-time objective intelligibility (STOI) [43], and the following three mean opinion scores: signal distortion (CSIG), background noise intrusiveness (CBAK), and overall signal quality (COVL) [44]. Table 2 compares the average PESQ, STOI, CSIG, CBAK, and COVL scores of the SE models evaluated on the test-clean dataset, according to the different training approaches.

As shown in the table, the standalone-SE model significantly improved the speech quality compared to noisy speech. Next, the SE models were excerpted from the pipeline trained using the different training approaches. It is shown in the third to fifth rows of the table that the SE models trained by the Joint-Straight, Joint-ASO, and Joint-Grad approaches achieved better speech quality than that of the noisy speech. However, all of the quality scores were lower than those of the standalone-SE model. The reason for these degraded results was that the conventional training approaches focused on improving the ASR performance rather than the SE performance.

In contrast, the SE model trained by Joint-Token and the proposed training approach achieved higher speech quality scores compared to the other three SE models trained by the conventional joint training approaches. Moreover, they were even better than those of the standalone-SE model. This was because the proposed training approach attempted to enhance the speech for better speech recognition, which resulted in better speech quality. Specifically, the proposed training approach significantly improved the speech intelligibility, as measured by PESQ, compared to Joint-Token, while the other speech quality scores of the proposed training approach were comparable to those of Joint-Token.

### 5.3. Discussion of Performance Contribution According to Different Losses

This ablation study examined the effectiveness of the proposed training approach according to different combinations of losses on the average WERs, as shown in Table 3. Note that the ASR model in the first row of the table corresponds to the ASR model trained by Joint-Grad, which showed the lowest WER among all of the conventional approaches, except for Joint-Token, as shown in Table 1. The second to the last rows present the WERs based on the loss functions used in the proposed training approach. The results in the second row present the effects of applying the combination of the SE and ASR encoder losses, $L_{NSNR}$ and $L_{Enc}$, on the ASR performance. Unfortunately, this loss combination increased the WER compared to Joint-Grad. In contrast, it is shown in the third row in the table that the tokenizer loss, $L_{Tokenizer},$ contributed to a marked reduction in the average WER, which corresponded to the Joint-Token training approach. Next, the proposed CBPC loss, $L_{CBPC}$, was combined with the previous three losses, resulting in lower WERs than those found in the case without combining $L_{CBPC}$, as shown in the fourth row of the table. Finally, all of the losses were combined to train the SE model. As shown in the last row of the table, this combination provided the lowest WER of all of the different loss combinations. This was because $L_{CBPC}$ mitigated the overfitting issue on the hard samples across the inter-cluster and $L_{infoNCE}$ improved the separability of the samples within the intra-cluster.

Table 4 compares the average speech quality scores of the SE models evaluated on the test-clean dataset according to different combinations of losses. As shown in the table, upon the integration of the tokenizer loss, all speech quality scores were improved compared to those of the standalone-SE model, which showed the highest speech quality scores among all of the conventional approaches, as shown in Table 2. However, applying the proposed CBPC loss function, $L_{CBPC}$, reduced the speech quality scores slightly. Finally, the proposed training approach using the combination of all losses achieved comparable CSIG, CBAK, and COVL scores to Joint-Token, but a higher PESQ score than Joint-Token, which confirms that PESQ is a metric that is related to ASR performance [45].

## 6. Conclusions

In this paper, the CBPC loss was proposed for noise-robust ASR in a joint training framework. To this end, a pipeline was constructed by using SE and ASR models. In this pipeline, an acoustic tokenizer leveraged the linguistic information from the ASR model to the SE model. The acoustic tokenizer took the outputs of the ASR encoder and provided a pseudo-label through K-means clustering. Then, to mitigate the problem of overfitting on hard samples across the inter-cluster, the proposed CBPC loss function was used to train the acoustic tokenizer. In addition, the infoNCE loss function was combined into the proposed CBPC loss function to improve the intra-cluster separability of the samples.

The WER of the ASR model trained using the proposed training approach was evaluated on a noisy LibriSpeech dataset and compared with those of ASR models trained using conventional training approaches, including MCT, MCT+standalone-SE, and four different joint training approaches. The results revealed that the ASR model trained by the proposed training approach with the CBPC loss function achieved the lowest WER among all of the compared models. In particular, the average WER of the ASR model trained using the proposed training approach was relatively reduced by 15.39% and 3.82% compared to those of the MCT and Joint-Token models, respectively. Next, the speech quality scores of the SE models were compared according to the different training approaches. Consequently, it was also observed that the proposed training approach provided the highest speech quality scores compared to the other approaches.

An ablation study was also conducted to investigate the effects of different combinations of loss functions used in the proposed training approach on the WER and speech quality scores. As a result, the combination of all loss functions, such as the tokenizer loss, CBPC loss, and infoNCE loss, provided the lowest WER and highest speech quality scores, except for CBAK.

In this work, the output vectors from the ASR encoder were clustered, and their cluster indices were used for the target labels of the acoustic tokenizer. As a result, after training the acoustic tokenizer using contrastive loss, there might be some mismatch between the target labels and the outputs of the acoustic tokenizer, as in metric learning [46,47]. In future studies, K-means clustering should be implemented, along with the acoustic tokenizer, to address this mismatch, which is expected to further improve the ASR and SE performance.

## Figures and Tables

**Table 1:** Comparison of the average word error rates (WERs) (%) of the ASR models according to different training approaches on the noisy LibriSpeech dataset.

Training Approach	Dev		Test		Average
	Clean	Other	Clean	Other	
MCT-noisy	22.77	23.18	22.95	23.41	23.08
+standalone-SE	28.94	29.03	29.38	29.14	29.12
MCT-all	22.61	22.74	22.68	22.82	22.71
Joint-Straight	22.39	22.51	22.40	22.64	22.49
Joint-ASO	22.31	22.42	22.37	22.58	22.42
Joint-Grad	20.11	20.89	20.88	20.98	20.72
Joint-Token	19.86	20.40	20.28	20.67	20.30
Proposed	**19.14**	**19.85**	**19.48**	**19.63**	**19.53**

**Table 2:** Comparison of the average perceptual evaluation of speech quality (PESQ), short-time objective intelligibility (STOI), and mean opinion scores, such as signal distortion (CSIG), background noise intrusiveness (CBAK), and overall signal quality (COVL), of the SE models, according to the different training approaches on the noisy LibriSpeech dataset.

Training Approach	PESQ	STOI	CSIG	CBAK	COVL
Noisy	1.7256	0.6967	1.8457	1.1615	1.3937
+standalone-SE	2.6512	0.8277	2.9671	2.5482	2.3410
Joint-Straight	2.4872	0.7504	2.8081	2.1950	2.1725
Joint-ASO	2.5871	0.7888	2.8213	2.3119	2.2647
Joint-Grad	2.5531	0.7719	2.8001	2.2964	2.2581
Joint-Token	2.6653	**0.8311**	3.1204	**2.5684**	**2.4509**
Proposed	**2.6802**	**0.8311**	**3.1275**	2.5653	2.4507

**Table 3:** Ablation study on the effectiveness of different loss combinations in the proposed training approach, measured as WER (%) (√ = applied to the proposed training approach).

Training Approach	Loss Function					Dev		Test		Average
	$L_{NSNR}$	$L_{Enc}$	$L_{Tokenizer}$	$L_{CBPC}$	$L_{infoNCE}$	Clean	Other	Clean	Other	
Joint-Grad						20.11	20.89	20.88	20.98	20.72
Proposed training	√	√				22.58	22.78	22.74	23.15	22.81
	√	√	√			19.86	20.40	20.28	20.67	20.30
	√	√	√	√		19.26	20.04	19.85	20.10	19.81
	√	√	√	√	√	**19.14**	**19.85**	**19.48**	**19.63**	**19.53**

**Table 4:** Ablation study on the effectiveness of different loss combinations in the proposed training approach, measured as PESQ, STOI, CSIG, CBAK, and COVL (√ = applied to the proposed training approach).

Training Approach	Loss Function					PESQ	STOI	CSIG	CBAK	COVL
	$L_{NSNR}$	$L_{Enc}$	$L_{Tokenizer}$	$L_{CBPC}$	$L_{infoNCE}$					
Noisy						1.7256	0.6967	1.8547	1.1615	1.3937
standalone-SE						2.6512	0.8277	2.9671	2.5482	2.3410
Proposed training	√	√				2.6500	0.8221	2.9595	2.5410	2.3387
	√	√	√			2.6653	**0.8311**	3.1204	**2.5684**	**2.4509**
	√	√	√	√		2.6638	0.8302	3.1192	2.5651	2.4472
	√	√	√	√	√	**2.6802**	**0.8311**	**3.1275**	2.5653	2.4507

## Data Availability

Publicly available datasets were used. LibriSpeech: https://www.openslr.org/12 (accessed on 3 March 2024). Deep noise suppression challenge: https://github.com/microsoft/DNS-Challenge/tree/interspeech2020/master (accessed on 3 March 2024).
